# Single-molecule FRET observes opposing effects of urea and TMAO on structurally similar meso- and thermophilic riboswitch RNAs

**DOI:** 10.1093/nar/gkad866

**Published:** 2023-10-19

**Authors:** Qian Hou, Surajit Chatterjee, Paul E Lund, Krishna C Suddala, Nils G Walter

**Affiliations:** Single Molecule Analysis Group, Department of Chemistry, University of Michigan, Ann Arbor, MI 48109, USA; Tri-Institutional PhD Program in Chemical Biology, Weill Cornell Medicine, The Rockefeller University, Memorial Sloan Kettering Cancer Center, NY, NY 10021, USA; Single Molecule Analysis Group, Department of Chemistry, University of Michigan, Ann Arbor, MI 48109, USA; Department of Physics, Case Western Reserve University, Cleveland, Ohio 44106, USA; Single Molecule Analysis Group, Department of Chemistry, University of Michigan, Ann Arbor, MI 48109, USA; Single Molecule Analysis Group, Department of Chemistry, University of Michigan, Ann Arbor, MI 48109, USA; Laboratory of Molecular Biology, National Institute of Diabetes and Digestive and Kidney Diseases, NIH, Bethesda, MD 20892, USA; Single Molecule Analysis Group, Department of Chemistry, University of Michigan, Ann Arbor, MI 48109, USA

## Abstract

Bacteria live in a broad range of environmental temperatures that require adaptations of their RNA sequences to maintain function. Riboswitches are regulatory RNAs that change conformation upon typically binding metabolite ligands to control bacterial gene expression. The paradigmatic small class-I preQ_1_ riboswitches from the mesophile *Bacillus subtilis* (*Bsu*) and the thermophile *Thermoanaerobacter tengcongensis* (*Tte*) adopt similar pseudoknot structures when bound to preQ_1_. Here, we use UV-melting analysis combined with single-molecule detected chemical denaturation by urea to compare the thermodynamic and kinetic folding properties of the two riboswitches, and the urea-countering effects of trimethylamine *N*-oxide (TMAO). Our results show that, first, the *Tte* riboswitch is more thermotolerant than the *Bsu* riboswitch, despite only subtle sequence differences. Second, using single-molecule FRET, we find that urea destabilizes the folded pseudoknot structure of both riboswitches, yet has a lower impact on the unfolding kinetics of the thermodynamically less stable *Bsu* riboswitch. Third, our analysis shows that TMAO counteracts urea denaturation and promotes folding of both the riboswitches, albeit with a smaller effect on the more stable *Tte* riboswitch. Together, these findings elucidate how subtle sequence adaptations in a thermophilic bacterium can stabilize a common RNA structure when a new ecological niche is conquered.

## Graphical Abstract

**Graphical Abstract:**
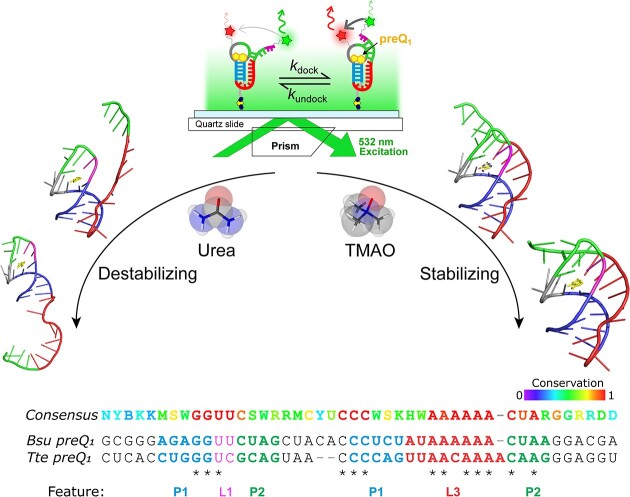

## Introduction

**Figure 1.**
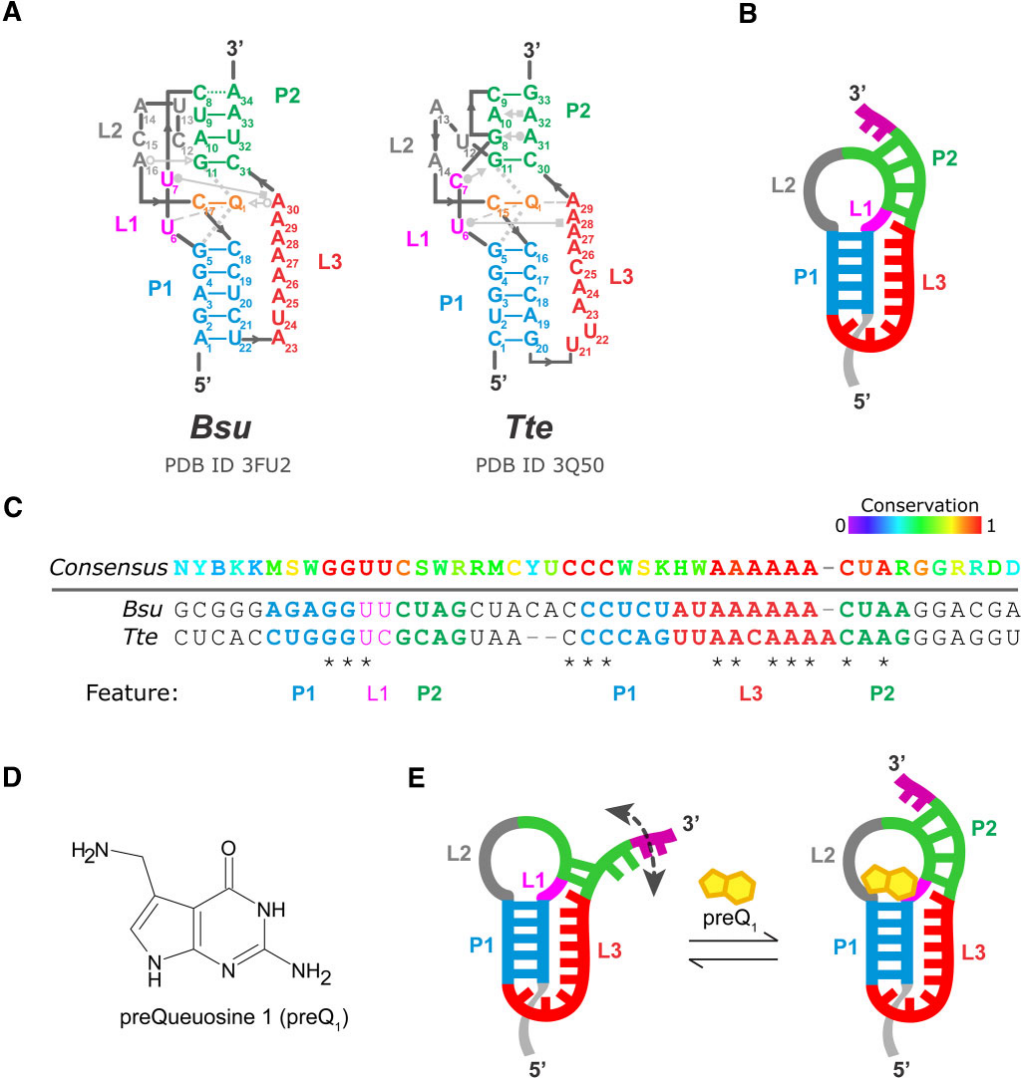
Sequence and structural comparison of the mesophilic (*Bsu*) and thermophilic (*Tte*) preQ_1_ riboswitches. (**A**) Secondary structure of the *Bsu* and *Tte* aptamer domains showing a subset of the key tertiary interactions in the Leontis-Westhof nomenclature (19). (**B**) Schematic representation of the secondary structure of the riboswitch pseudoknot. (**C**) *Bsu* and *Tte* preQ_1_ riboswitches sequence conservation. Top, consensus sequence (8) using IUPAC ambiguity codes. (**D**) Chemical structure of the preQ_1_ (7-aminomethyl-7-deazaguanine) ligand. (**E**) preQ_1_-induced structural rearrangement (‘docking’) of the pseudoknot where the P2 helix becomes fully formed.

Riboswitches are regulatory non-coding RNA motifs found in the 5′-untranslated regions (UTRs) of many bacterial messenger RNAs (mRNAs) (1–4) and are composed of two structural domains: a highly conserved aptamer and a downstream expression platform. Selective binding of cellular metabolites (ligands) by the aptamer domain modulates downstream RNA folding and regulates gene expression (4,5) through either transcription attenuation (transcriptional riboswitches) or inhibition of translation initiation (translational riboswitches) (6,7). The class-I preQ_1_ (preQ_1_-I) riboswitch has one of the smallest known aptamer domains and recognizes the ligand 7-aminomethyl-7-deazaguanine (preQ_1_) for gene regulation. The preQ_1_-I aptamers from the mesophilic *Bacilus subtilis* (*Bsu*) and the thermophilic *Thermoanaerobacter tengcongensis* (*Tte*) have highly similar aptamer sequences and structural features (Figure 1A–C). Ligand binding to these riboswitches promotes the formation of compact H-type pseudoknot structures (Figure 1D, E). Both pseudoknots contain stem P1 (5 base pairs, bp), loop L1 (2 bp), stem P2 (4 bp), loop L2 and loop L3 with some variation in sequence (Figure 1C) (8). The riboswitches are functionally distinct, operating through transcription termination (*Bsu*) and translational repression (*Tte*) (9,10). In the *Bsu* riboswitch, formation of ligand-bound pseudoknot prevents an anti-terminator hairpin from forming and therefore leads to transcription termination (9). In contrast, folding of the *Tte* riboswitch RNA into the pseudoknot structure sequesters the first two nucleotides of the Shine-Dalgarno (SD) sequence, thereby downregulating translation initiation (10).

Previous comparative studies of the isolated *Bsu* and *Tte* riboswitches (formally, their aptamer domains) have demonstrated that both RNAs adopt similar conformational ensembles under near-physiological buffer conditions with 1 mM Mg^2+^ (11). Using single molecule fluorescence resonance energy transfer (smFRET) to monitor the conformational dynamics revealed that both riboswitches exhibit an ‘undocked’ pre-folded conformation wherein the 3′ A-rich tail transiently interacts with the P1-L1 stem-loop to form P2 in the ‘docked’ folded conformation. In the presence of preQ_1_, P2 is further stabilized, and the folded state becomes more favorable (Figure 1E). Despite these general conformational similarities between the two RNAs, the ligand-free *Tte* aptamer shows characteristically slower dynamics and a higher population of the docked state than the *Bsu* aptamer, indicating a generally higher degree of structural compactness (11). Computational modeling further indicates that preQ_1_ binding to the *Bsu* and *Tte* aptamers preferentially occurs through conformational selection and induced fit mechanisms, respectively, suggesting distinct folding pathways (11). The two folding pathways can be distinguished by the timing of ligand binding relative to full pseudoknot folding. In the conformational selection pathway, ligand binding to a preformed RNA pocket occurs late, while 3′ A-rich tail docking and the formation of P2 occur simultaneously with ligand binding. In contrast, in the induced fit pathway an early ligand-binding event initiates the formation of the remaining tertiary contacts. Such distinctions measured at room temperature correlate with the distinct environments wherein the two bacteria exist; the *Tte* preQ_1_ riboswitch originates from a thermophilic bacterium that grows optimally at a high temperature of 75°C (12) and thus would be expected to be more thermostable than the mesophilic *Bsu* riboswitch with an optimum temperature of 35°C. However, it is unknown whether these two RNAs exhibit differences in their stabilities to thermal and chemical denaturation, given the high similarity of their sequences and tertiary structures. Furthermore, a more detailed understanding of the differences in structural stability between the two aptamers in relation to their solvent environment is also lacking.

Previous biophysical studies have heavily focused on the influential role of mono- and divalent metal cations in the folding of polyanionic nucleic acids (11,13–15). Recently, osmolytes have been shown to also impact RNA folding equilibria through their interaction with nucleobases, ribose sugars and phosphate groups (16–18). Osmolytes are small water-soluble organic molecules utilized by certain cells to regulate osmotic pressure (20). Depending on the chemical nature of an osmolyte, it can either promote or inhibit the folding of nucleic acids and proteins (21,22). Therefore, while minimizing fluctuations in cellular osmotic pressure, it is also critical for cells to maintain proper intracellular concentrations of both structurally destabilizing and stabilizing osmolytes so that their opposing effects on nucleic acid and protein stabilities are counterbalanced. One such osmolyte, urea, is one of the most widely used denaturants of both proteins and nucleic acids (23–25), whereas trimethylamine *N*-oxide (TMAO) is known for its abilities to promote biopolymer folding (24,26–28) and counteract the denaturing effect of urea (16,18,24,26,29). Due to the opposing effects of urea and TMAO on biomolecule stability, we hypothesize that they can be used in combination to finely adjust the degree of conformational transition. By employing smFRET microscopy, we will be able to extract the energetic and kinetic characteristics of structurally similar *Bsu* and *Tte* riboswitch aptamers.

In this study, we investigate the thermodynamic stability of the two riboswitch RNAs by directly probing their heat denaturation, which is detected by changes in UV absorption. Our results demonstrate that the *Tte* aptamer exhibits higher thermal stability compared to the *Bsu* aptamer, and this stability is significantly enhanced by ligand binding. By combining urea-based denaturation and smFRET, we find that urea has a more pronounced impact on the conformational dynamics of the *Bsu* riboswitch compared to the *Tte* riboswitch. Furthermore, we observe a strong interplay among urea, TMAO, and Mg^2+^ ions, where TMAO and Mg^2+^ effectively counteract the denaturing effects of urea on both RNAs. Our data show that TMAO exerts a stronger protective effect against urea-induced unfolding of the *Bsu* riboswitch, while its influence on the more stable *Tte* riboswitch is comparatively less prominent. Finally, *m*-value analyses of the transition kinetics further support that, upon solvent perturbation, the *Tte* riboswitch displays a higher structural stability than *Bsu*. We anticipate that monitoring structural perturbations induced by osmolytes using smFRET microscopy has the potential to serve as a generalizable tool for discerning differences in the conformational free energy landscapes of structurally similar RNAs.

## Materials and methods

### RNA preparation for melting curve studies

*Bsu* and *Tte* preQ_1_ riboswitch aptamers for melting experiments were generated by *in vitro* transcription using T7 RNA polymerase (RNAP) prepared in-house as previously described by He *et al.* (30) T7 RNAP was expressed in BL21 with N-terminal His-tag, and purified in 250 mM NaCl preventing co-purification of other proteins. Affinity-based purification was achieved by a hybrid batch/gravity-flow procedure. *In vitro* transcription reactions were designed based on previously described conditions with minor modifications. DNA oligonucleotides from IDT were purified by 20% denaturing urea polyacrylamide gel electrophoresis and excised under UV-shadowing with a 312 nm lamp. The DNA templates (*Tte*: 5′-CCC TTG TTT TGT TAA CTG GGG TTA CTG CGA CCC AGG ACC TAT AGT GAG TCG TAT TAA ATT-3′; 5′-AAT TTA ATA CGA CTC ACT ATA GG-3′, *Bsu*: 5′- CCT TAG TTT TTT ATA GAG GGT GTA ACT AGA ACC TCT GCC TAT AGT GAG TCG TAT TAA ATT -3′; 5′-AAT TTA ATA CGA CTC ACT ATA GG-3′) were eluted from the gel, ethanol precipitated and resuspended in nuclease free water. 300 μl transcription reactions containing 10 μM T7-*Bsu* (or *Tte*) duplex template, 120 mM HEPES–KOH (pH 7.6 at 22°C), 0.01% (v/v) Triton X-100, 30 mM MgCl_2_, 7.5 mM of each NTP, 40 mM DTT, 2 mM spermidine trihydrochloride, 0.2 mg/ml T7 RNAP, and 0.01 U/μl inorganic pyrophosphatase (MP Biomedicals) were incubated in a circulating water bath at 37°C for 16–18 hours, and then mixed with an equal volume of 2× gel loading buffer (95% (v/v) formamide, 18 mM EDTA, 0.025% [w/v] each of SDS, bromophenol blue, and xylene cyanol) to stop the reaction. The reaction with loading buffer was heated at 90°C for 3 min and then snap cooled on ice. The RNA transcripts were gel purified as described for DNA above.

### UV–Vis melting studies

Melting experiments were conducted on a Beckman DU® 640B spectrophotometer with a Beckman High Performance Temperature Controller, a Transport accessory, a *T*_m_ Six-Cell Holder, and 1 cm path length quartz cuvettes (Beckman Coulter, 523878). Tris-based buffers were replaced with sodium phosphate as buffering salt, and ionic strength was adjusted to match 1× smFRET buffer. A typical sample contained 0.3 μM RNA construct (*Bsu* or *Tte*), 10 mM sodium phosphate (pH 7.0 at 22°C), 100 mM NH_4_Cl, and with or without 1 mM MgCl_2_ depending on the condition being tested. The complete sequences for RNA constructs used in this study are *Tte*: 5′-ggucCUGGGUCGCAGUAACCCCAGUUAACAAAACAAGGG-3′ and *Bsu*: 5′-ggcAGAGGUUCUAGUUACACCCUCUAUAAAAAACUAAGG-3′.

Samples were refolded by heating at 90°C for 3 min, at 70°C for 3 min, at 60°C for 3 min, and finally allowed to cool to room temperature over 20 min. For experiments done with saturating ligand, 0.45 μM preQ_1_ was added to the refolded RNA (1.5:1 ligand:RNA final concentration ratio). 325 μl sample was transferred to each cuvette and tightly stoppered. *A*_260_ was monitored with a 0.5 s read averaging time, and *A*_340_ or *A*_320_ was used for background correction. Temperature was increased at a rate of 1°C/min between 10 and 22°C with a reading made every 1°C, then at a rate of 0.5°C/min between 22 and 75°C with a reading made every 0.5°C, then at a rate of 1°C/min between 75 and 95°C with a reading made every 1°C. Only denaturation transition curves were collected, and each condition was repeated four times. The melting temperature for each apparent transition was determined as described previously from the second derivative of the absorbance versus temperature plot (31).

### Preparation of labeled RNA for smFRET experiments

The smFRET constructs for *Bsu* and *Tte* preQ_1_ riboswitch aptamers were prepared as previously described (11). Briefly, the RNAs were chemically synthesized by Dharmacon, Inc. (Fayette, Colorado) with modifications as follows: 5′-biotinylated, 3′-DY547 labeled and 5-aminoallyl uridine (5NU) labeled at position U12 (*Tte*) and U13 (*Bsu*). The RNAs were first deprotected following the manufacturer's instructions and then labeled with Cy5-NHS ester (GE Healthcare). One dye pack was used for labeling one construct. The dye pack was dissolved in 30 μl of DMSO and used to label 3.4 nmol RNA in a total reaction volume of 50 μl containing 0.1 M sodium bicarbonate buffer (pH 8.7). The mixture was then incubated and tumbled at room temperature in the dark for 4 h. Excess free dye was removed by using a Nap-5 gel filtration column (GE Healthcare). The RNAs were collected, ethanol precipitated and dissolved in autoclaved, deionized water (11). The complete sequences for the RNA constructs used in smFRET experiments were *Bsu*: 5′-biotin-ugcgggAGAGGUUCUAGCUACACCCUCUAUAAAAAACUAAGG-3′ and *Tte*: 5′-biotin-ucacCUGGGUCGCAGUAACCCCAGUUAACAAAACAAGGG-3′.

### Single-molecule FRET experiments

We assembled a microfluidic channel in between a clean quartz slide and a glass coverslip as described by previous smFRET studies (32). We then coated the quartz slide with biotinylated-BSA followed by streptavidin. The RNA was folded in 1X smFRET buffer (50 mM Tris–HCl, 100 mM KCl, pH 7.5) in the absence of Mg^2+^, by heating at a low concentration (20–30 pM) at 90°C for 1 min, snap-cooling on ice for 30 s, then allowing to slowly reach room temperature for over 15 min. 100–200 μl of the heat-annealed RNA was flowed into the microfluidic channel and incubated for 5 min. The surface bound streptavidin was able to capture the 5′-biotin on the RNA construct and ensure the surface immobilization of the RNA. The unbound free RNA was washed away using 1× smFRET buffer (±Mg^2+^ depending on the experiment). Osmolyte titration was performed with or without 1 mM Mg^2+^, as indicated, and in the presence of 100 nM preQ_1_. An oxygen scavenging system (OSS) containing 5 mM protocatechuic acid (PCA), 50 nM protocatechuate-3,4-dioxygenase (PCD), and 2 mM Trolox (6-hydroxy-2,5,7,8-tetramethylchroman-2-carboxylic acid) was introduced into the microfluidic channel serving the purpose of extending the life of fluorophores and reducing fluorophore photoblinking events (32,33). The experiments were performed on a prism*-*based total internal reflection fluorescence (TIRF) microscope. A 532 nm laser was used to excite DY547, and emission from both DY547 and Cy5 was recorded concurrently using an intensified charge-coupled device camera (ICCD, I-Pentamax, Princeton Instruments) at 60 ms time resolution (11). We processed raw movie files using IDL (Research Systems) to extract smFRET time traces that were analyzed using MATLAB (The Math Works) scripts. We manually selected smFRET time traces meeting the following criteria: single-step photobleaching, a trajectory length of at least 100 camera frames before bleaching, a signal-to-noise ratio of >4:1, and a total fluorescence intensity (donor + acceptor) of >300 (arbitrary units) (11). *I*_D_ and *I*_A_ represent the background corrected intensities of DY547 (donor) and Cy5 (acceptor) fluorophores, and the FRET efficiency was calculated as E_FRET_ = I_A_/(I_D_ + I_A_). FRET efficiencies observed in first 100 frames from each trace were combined to generate FRET distribution histograms using MATLAB. Dynamic time traces were idealized with a two-state model according to Hidden-Markov Modeling (HMM) using a segmental k-means algorithm in QuB software. Transition Density Plots (TDPs) and Transition Occupancy Density Plots (TODPs) were then generated using MATLAB (34). A summary of the total number of molecules collected under each experimental for smFRET analysis is provided in Supplementary Table S1.

### Rate constant analysis from smFRET data

Dwell times in the undocked and docked states were extracted from all the idealized traces, and the cumulative dwell time distributions were fit with an exponential function of the form $y = A( {1 - {e}^{ - k\tau }} )$ or $y = {A}_1( {1 - {e}^{ - {k}_1\tau }} ) + {A}_2( {1 - {e}^{ - {k}_2\tau }} )$ to obtain the rate constants *k*_dock_ and *k*_undock_, respectively, as described previously (31). Exponential fitting was performed in MATLAB. In cases where the 95% confidence interval for the fitting parameters contained negative values, or when there were too few data points, the double-exponential fit was rejected in favor of a single-exponential fit. Whenever data were better fit with a double-exponential function, more than one underlying process with each a characteristic rate constant appears to be occurring, involving, e.g. formation of an intermediate. To compare our data across conditions, the average rate constant of the double-exponential fits was calculated as the weighted average of the two rate constants (*A*_1_*k*_1_ + *A*_2_*k*_2_). A summary of the number of dynamic traces that contributed to the rate constant determinations for each experimental condition and whether a single- or double-exponential fit was used is provided in Supplementary Table S2. The error associated with the measured rate constants was estimated by bootstrap fitting on 1000 sets of replicate data that were chosen by sampling *M* traces from traces in original dataset with replacement, where *M* is equal to the number of traces present in the original dataset. The standard deviation of the resulting 1000 replicate fit-coefficients is reported as the error of measured rate constant (31).

### *m*-value analysis of smFRET data

The change in the Gibbs free energy of docking ($\Delta {\mathrm{G}}{^\circ }_{dock}$) for the riboswitch aptamers in the presence of increasing concentrations of osmolyte was determined from the smFRET distribution histograms. After fitting the FRET distribution to the sum of two Gaussians (the first for the high FRET population and the second for the mid-FRET population), the area under the curve (AUC) for each Gaussian was used to calculate $\Delta {\mathrm{G}}{^\circ }_{dock}$ with the following equation: $\Delta {\mathrm{G}}{^\circ }_{dock} = \ - RTln{{\mathrm{K}}}_{eq} = \ - RTln( {AU{C}_{high\ FRET}/AU{C}_{mid\ FRET}} )$, with *T* = 20°C. The error of $\Delta {\mathrm{G}}{^\circ }_{dock}$ at each osmolyte concentration was estimated again through bootstrapping. Briefly, $\Delta {\mathrm{G}}{^\circ }_{dock}$ was determined for 1000 sets of replicate data, where each set is chosen by sampling *M* traces from traces in the original dataset with replacement, and where *M* is equal to the number of traces present in the original dataset. The standard deviation of the resulting 1000 replicates is reported as the error of${\mathrm{\ }}\Delta {\mathrm{G}}{^\circ }_{dock}$ at a given osmolyte concentration. The slope of the linear fit (*m*-value) of $\Delta {\mathrm{G}}{^\circ }_{dock}$ versus osmolyte concentration was determined in OriginLab, where the error of the fitting parameter is taken as the error of the *m*-value.

## Results

### UV–Vis melting reveals higher thermal stability for the *Tte* than the *Bsu* riboswitch

Due to its origin from a thermophilic bacterium, the *Tte* riboswitch is expected to be more thermostable than the mesophilic *Bsu* riboswitch. However, there is a lack of data providing a side-by-side comparison of their melting temperatures or thermal stabilities. Therefore, to directly assess and compare the relative stabilities of the two riboswitch pseudoknots, we conducted UV-Vis melting studies in the absence or presence of 1 mM Mg^2+^ and of a 1.5-fold molar excess of cognate ligand preQ_1_ over RNA (0.45 μM preQ_1_, considerably above the known ligand binding affinities in the nanomolar range (11), and 0.3 μM RNA). Notably, under all conditions tested, the temperature required to fully denature the *Tte* aptamer was found to be approximately 10–20°C higher than that of *Bsu* (Table 1). The highly thermally stable P1 stem of the *Tte* riboswitch provides the structural basis for the *Tte* riboswitch to function at elevated environmental temperatures.

**Table 1.** The melting temperatures of stems P2 and P1 of the *Tte* and *Bsu* riboswitches in the presence and absence of preQ_1_ and Mg^2+^ as indicated. Under equivalent experimental conditions, the P2 stem of the *Bsu* riboswitch does not show a separate melting transition

	-preQ_1_, -Mg^2+^ (ºC)	-preQ_1_, +Mg^2+^ (ºC)	+preQ_1_, -Mg^2+^ (ºC)	+preQ_1_, +Mg^2+^ (ºC)
**P2**				
*Tte*	40 ± 2	48 ± 1	53 ± 1	57 ± 1
*Bsu*				
**P1 (& P2 for *Bsu*)**				
*Tte*	66 ± 1	67 ± 1	74 ± 4	76 ± 2
*Bsu*	54 ± 2	56 ± 1	55 ± 1	55 ± 1

**Figure 2.**
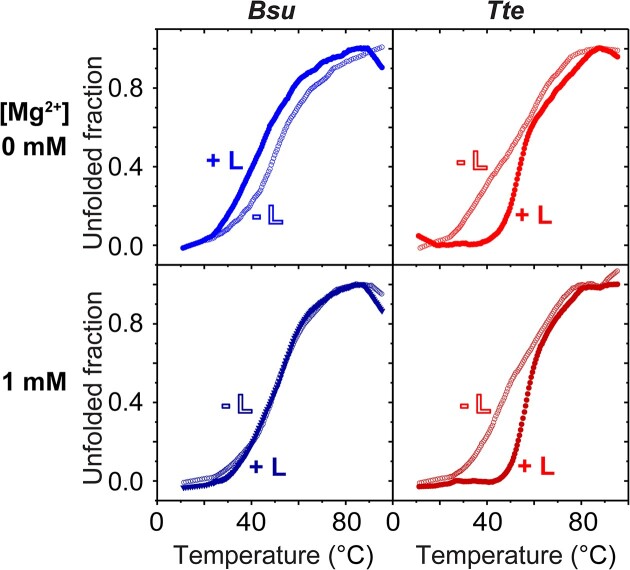
Probing the thermal stabilities of the structurally similar *Bsu* and *Tte* riboswitches. Melting curve analysis of the *Bsu* and *Tte* riboswitches in the absence and presence of Mg^2+^ and preQ_1_ ligand (L) as indicated.

In addition to displaying higher thermal stability, the *Tte* riboswitch aptamer exhibited stepwise melting of P2 and P1 helices, whereas the *Bsu* riboswitch aptamer displayed a single melting transition. Specifically, in the absence of Mg^2+^ and preQ_1_, the *Tte* riboswitch showed two distinct melting transitions at approximately 40ºC and 66ºC (Figure 2, Table 1). These transitions were previously assigned to melting of stem P2 (and L3) and stem-loop P1, respectively (31). The presence of 1 mM Mg^2+^ increased the melting temperature of P2 to approximately 48ºC, while the melting temperature of P1 remained unaffected. This stabilizing effect of Mg^2+^ is in line with the well-known ability of Mg^2+^ to enhance the stability of RNA tertiary interactions and pseudoknot folding (11). Recent structural analysis of the *Tte* riboswitch further underscored the significance of divalent ions in its folding process, as it revealed the presence of six divalent metal ion binding sites within both the apo and ligand-bound structures of *Tte* (35). On the other hand, upon addition of preQ_1_, we observed a significant increase in the melting temperatures of P2 and P1, regardless of the absence and presence of Mg^2+^ (Figure 2, Table 1). The *Tte* preQ_1_ riboswitch is thought to follow the induced fit pathway, where the preQ_1_ ligand binds to the RNA with high affinity, inducing pseudoknot folding (11,31). Therefore, the folding of *Tte* is highly dependent on the presence of the ligand, consistent with our experimental findings. Moreover, recent structures of *Tte* revealed the ligand-induced coaxial stacking of the P2 and P1 stems (35). This phenomenon provides an explanation for the ligand-induced increase in the melting temperatures of both P2 and P1, as the coaxial stacking contributes additional stability to the structure. In contrast, for the *Bsu* riboswitch, we observed only a single melting transition at ∼54ºC, which remained largely unaffected by the presence of Mg^2+^ and preQ_1_ (Figure 2, Table 1). Notably, *Bsu* does not exhibit a distinct gap in the thermal stability between P1 and P2, as the formation of P2 is an intrinsic characteristic of the RNA structure rather than being induced by the additional stability provided by ligand binding (13).

### smFRET reveals distinct conformational responses for the *Bsu* and *Tte* riboswitches in the presence of urea

**Figure 3.**
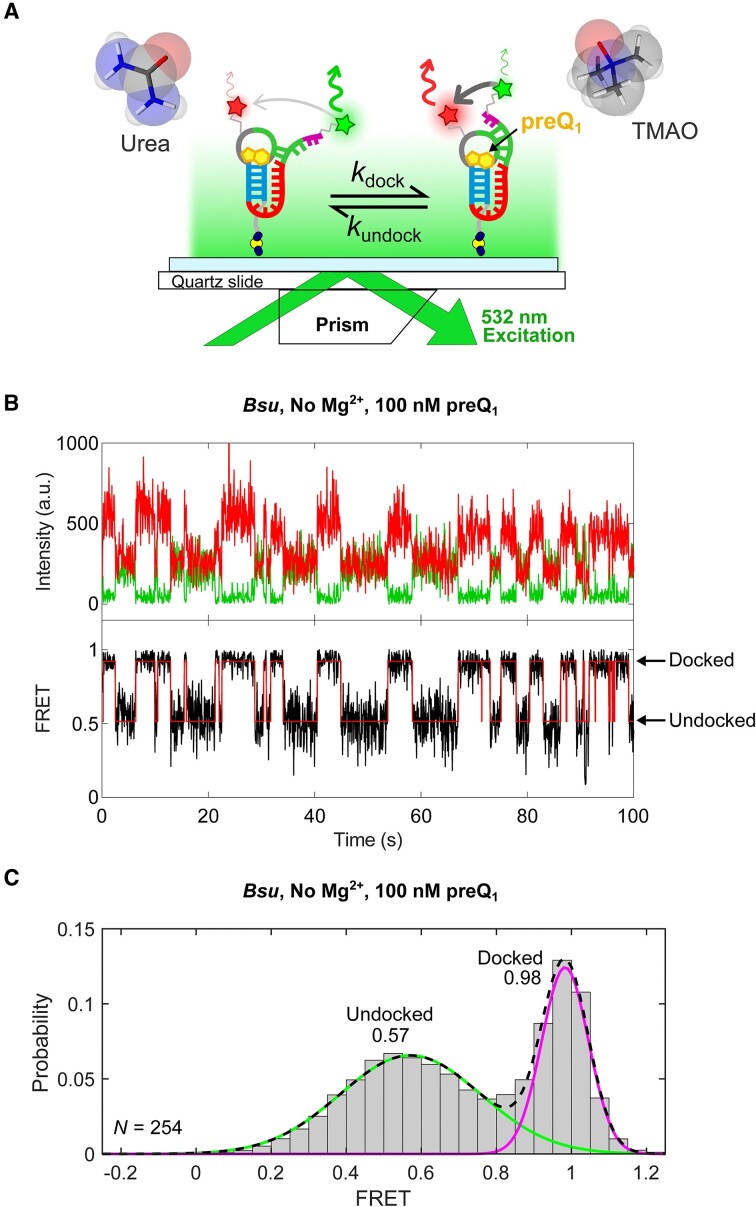
Single-molecule FRET probing of the relative structural stabilities of the *Bsu* and *Tte* riboswitches. (**A**) Schematic for smFRET-based assessment of osmolyte (urea/TMAO) impact on RNA folding through effects on docking/undocking kinetics of the two closely related preQ_1_ riboswitches. (**B**) Top, representative fluorescence intensity *vs* time trace showing anti-correlated changes in donor (Cy3) and acceptor (Cy5) intensities due to FRET during docking and undocking of helix P2 of the preQ_1_ riboswitch. Bottom, changes in the FRET efficiency calculated from the trace, observed here in the absence of Mg^2+^. (**C**) smFRET histogram constructed from all FRET values in the dataset observed over the first 100 observation frames, showing two major FRET states fitted with Gaussian distributions that yield mean FRET values as indicated. *N*, number of molecules.

**Figure 4.**
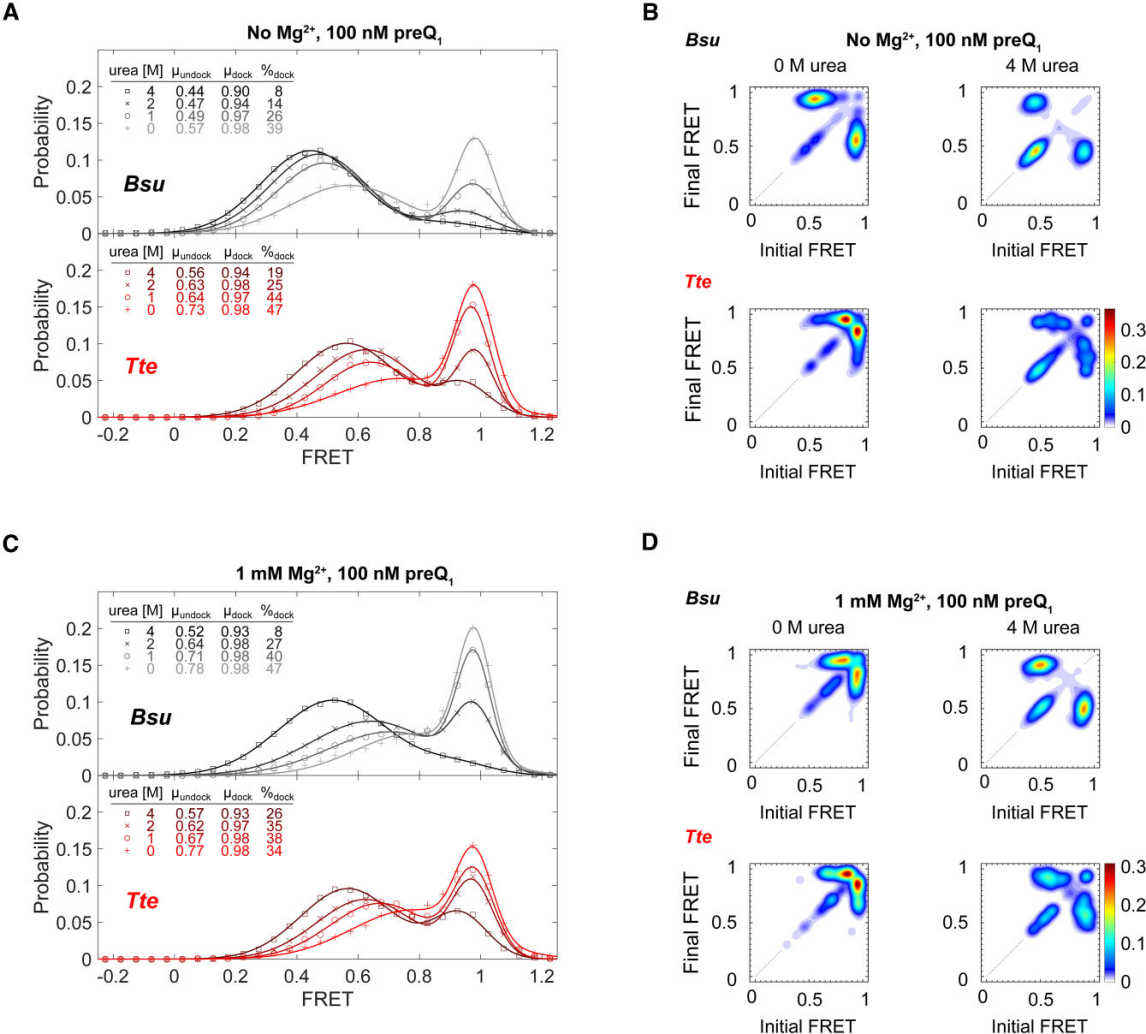
Urea destabilizes the folded conformation of the *Bsu* and *Tte* riboswitches. (**A**) FRET population distribution histograms of the *Bsu* (top panel) and *Tte* (bottom panel) riboswitches in the absence of Mg^2+^ with increasing urea concentration, showing shifts in the mid-FRET and high-FRET populations as quantified in the table. (**B**) Transition occupancy density plots (TODPs) for the *Bsu* (top) and *Tte* (bottom) riboswitches in the absence of Mg^2+^ showing the most common FRET transitions in the absence and presence of 4 M urea. (**C**, **D**) FRET population distribution histograms and TODPs as in panels a and b, respectively, but with 1 mM Mg^2+^ added. 100 nM preQ_1_ was used in all experiments to induce dynamic docking of the molecules.

While the ensemble UV–Vis melting analysis unveils the relative thermodynamic structural stabilities of the *Bsu* and *Tte* riboswitches, it lacks information on their folding kinetics. Previously, we used smFRET to study the P2 helix folding kinetics of the *Bsu* and *Tte* riboswitches in the presence of Mg^2+^ and various ligands that bind and stabilize the folded structure (11,13). To understand the kinetic origin of the observed differences in RNA stability, we therefore utilized smFRET to investigate the pseudoknot formation (i.e. docking) dynamics of these riboswitches in the presence of the osmolytes urea and TMAO (Figure 3A). We kept the preQ_1_ concentration at 100 nM for all experiments to enable observation of folding dynamics, a condition where the full P2 stem reversibly docks even in the absence of Mg^2+^. Accordingly, single molecule time trajectories demonstrate two-state folding kinetics with well-defined mid-FRET and high-FRET states in the absence of Mg^2+^ (Figure 3B; the molecule number for each smFRET experiment presented here—typically between ∼50 and 350—is reported in Table S1), consistent with previous assignments of the undocked and docked pseudoknot conformations, respectively (11,13). The corresponding FRET histogram for the *Bsu* riboswitch exhibits a mid-FRET state, representing the undocked conformation (mean FRET efficiency, μ_undock_ ∼0.57 with 61% population weight; Figure 3C), and a high-FRET state representing the docked conformation (μ_dock_ ∼0.98, 39%; Figure 3C). Notably, the mean FRET efficiency of the mid-FRET state, μ_undock_, shifts gradually to lower values with increasing concentration of urea to reach μ_undock_ ∼0.4 at 4 M urea, while in parallel the occupancy of the high-FRET state steadily decreases to only 8% with only a small reduction in μ_dock_ to 0.90 (Figure 4A, top panel). This shows that urea unfolds the docked pseudoknot conformation, as indicated by a decrease in percentage occupancy at μ_dock_. This is consistent with the ability of urea to denature RNA structures (16,25). Similarly, FRET histograms for the *Tte* riboswitch show a mid-FRET state of μ_undock_ ∼0.73 (53%), a high-FRET state at μ_dock_ ∼0.98 (47%), and display generally similar trends as the *Bsu* riboswitch with both mid-FRET values and high-FRET state occupancy decreasing in the presence of increasing urea concentrations (Figure 4A, bottom panel). However, the value of mid-FRET at 4 M urea remains relatively higher for *Tte* (μ_undock_ ∼0.6) than that for *Bsu* (μ_undock_ ∼0.4), suggesting less opening of the *Tte* riboswitch upon urea denaturation than the *Bsu* riboswitch despite their overall similar topology and sequence (Figure 1A).

From the smFRET traces, dwell times in the mid- and high-FRET states were extracted using a two-state Hidden Markov Model (HMM) and fit with single-exponential functions to estimate the rate constants of docking (*k*_dock_) and undocking (*k*_undock_), respectively. For *Bsu*, in the presence of 100 nM preQ_1_ but no Mg^2+^, *k*_dock_ decreases gradually from 1.35 s^−1^ in the absence of urea to 0.1 s^−1^ in the presence of 4 M urea (Supplementary Figure S1a). Conversely, *k*_undock_ increases from 0.5 s^−1^ in the absence of urea to 2.3 s^−1^ in the presence of 4 M urea (Supplementary Figure S1a). A similar trend was observed for the *Tte* riboswitch; *k*_dock_ decreases from 4.3 s^−1^ to 0.5 s^−1^ and *k*_undock_ increases from 1.2 s^−1^ to 11 s^−1^ from 0 to 4 M urea (the latter value approaching our 60 ms time resolution; Figure S1b). These observations suggest that, in the absence of Mg^2+^, urea destabilizes P2 formation by both slowing docking and accelerating undocking for both the *Bsu* and *Tte* riboswitches, similar to prior observations for an idealized tertiary structure docking system (18).

Transition-state analysis (Φ) is a valuable tool for studying the nature of the transition state, elucidating the origin of urea's destabilizing effect, and unraveling its influence on tertiary interactions along the folding pathway (13,36,37). In the case of urea denaturation, Φ allows us to compare the energetic impact of urea on the rate constant for docking with its effect on the equilibrium of docking. A Φ value of 0 would indicate that the interaction affected by urea has not formed in the transition state, while a Φ value of 1 would suggest that the interaction affected by urea fully forms in the transition state. A fractional value implies the partial formation of tertiary contacts during the transition state. Our data yield a Φ value of 0.63 for *Bsu* and Φ value of 0.49 for *Tte*, indicating that the tertiary interactions perturbed by urea partially form already in the transition state (Supplementary Table S3). Furthermore, these findings suggest that the docked state is more destabilized by urea than the transition state relative to the undocked state (13).

To further investigate the mechanism of the urea-induced unfolding of the two riboswitches, we generated transition occupancy density plots (TODPs). These plots, presented as heat maps (34), illustrate the fraction of molecules that undergo a particular transition between defined FRET states at least once during the observation period. With no Mg^2+^ but in the presence of preQ_1_, both riboswitches exhibit a negligible contribution from a static mid-FRET population. However, with increasing urea concentration, the occupancy of this population increases (as observed in the on-diagonal populations in Figure 4B). This suggests that an increasing number of molecules adopt an undocked conformation with minimal dynamic character (as far as observable at the time resolution of the experiment, 60 ms). Comparing the TODPs between *Tte* and *Bsu*, we observe that the *Tte* riboswitch displays a closer proximity between the two interconverting dynamic FRET states, indicating a more compact conformation than for the *Bsu* riboswitch (Figure 4B, left panels). Furthermore, at 4 M urea, the *Tte* riboswitch exhibits increased heterogeneity with multiple underlying FRET values (Figure 4B, right panels).

In the presence of both 1 mM Mg^2+^ and preQ_1_, urea has less impact on the FRET histograms of both riboswitches (Figure 4C), which is consistent with the stabilizing effect of Mg^2+^. The high-FRET populations of both the *Bsu* and *Tte* riboswitches still gradually decrease with increasing urea concentration, although not to the same extent as observed under the condition without Mg^2+^. In particular, the changes in the fractions of high-FRET population are less at 2 M urea when Mg^2+^ is present compared to its absence (Figure 4C). Furthermore, differences in (un)folding kinetics were observed between the two riboswitches. Specifically, the undocking rate constant *k*_undock_ of the *Bsu* riboswitch remains relatively unchanged upon addition of urea, whereas *k*_undock_ for the *Tte* riboswitch significantly increases (Supplementary Figure S1c, d). At the same time, the docking rate constant *k*_dock_ of the *Bsu* riboswitch decreases more profoundly than that of *Tte* (Supplementary Figure S1c, d). Based on transition-state analysis, an unchanged *k*_undock_ results in a Φ value of 1 for *Bsu*, indicating that the tertiary interactions perturbed by urea have already been fully established in the transition state upon addition of 1 mM Mg^2+^. TODPs provide additional insights into the differences in transition dynamics. In the presence of Mg^2+^ (with no urea), a significant Mg^2+^-induced compaction occurs, resulting in two closely spaced interconverting FRET states (compare Figure 4B and D). This finding is consistent with the mid-FRET state of the *Bsu* riboswitch shifting from μ_undock_ ∼0.57 to μ_undock_ ∼0.78 in the presence of Mg^2+^ (compare Figure 4A and C). In contrast, the *Tte* riboswitch exhibits a high mid-FRET value even without Mg^2+^ and preQ_1_, and this value remains largely unchanged upon the addition of Mg^2+^. Overall, Mg^2+^ sufficiently stabilizes the pseudoknot in both riboswitches, leading to increased resistance to urea denaturation. This observation is further supported by the relatively small fraction of static mid-FRET state molecules even at 4 M urea (compare Figure 4B and D).

### TMAO promotes riboswitch folding and counteracts urea-induced denaturation

The osmolyte TMAO is known to promote the folded conformation of RNA due to its unfavorable interaction with exposed phosphate groups of the RNA backbone (28) and is thus expected to counteract urea (18). To further compare the folding properties of the *Bsu* and *Tte* riboswitches, we studied the effects of TMAO (in the presence of 100 nM preQ_1_) on RNA folding, first in the absence of urea. With no Mg^2+^, in case of the *Bsu* riboswitch, we observed a gradual increase in the mean FRET efficiency of the mid-FRET state from μ_undock_ ∼0.67 in the absence of TMAO to ∼0.83 in the presence of 2 M TMAO, indicating that the osmolyte further compacts the undocked *Bsu* riboswitch (Supplementary Figure S2a). This effect is smaller (from 0.75 to 0.87) in the presence of both preQ_1_ and 1 mM Mg^2+^ due to the overall compaction in the *Bsu* pseudoknot already induced by the divalent metal ion (Supplementary Figure S2b). In contrast, the effects of TMAO on the *Tte* riboswitch are insignificant (data not shown) since the *Tte* riboswitch is already tightly folded in the presence of only 100 nM preQ_1_, regardless of the presence or absence of Mg^2+^. Furthermore, kinetic analysis of the smFRET time traces reveals an increase in *k*_dock_ for the *Bsu* riboswitch with increasing TMAO concentration. This is in contrast to the relatively small effect on *k*_undock_ of *Bsu* (Supplementary Figure S3) and either rate constant of the *Tte* riboswitch (data not shown).

**Figure 5.**
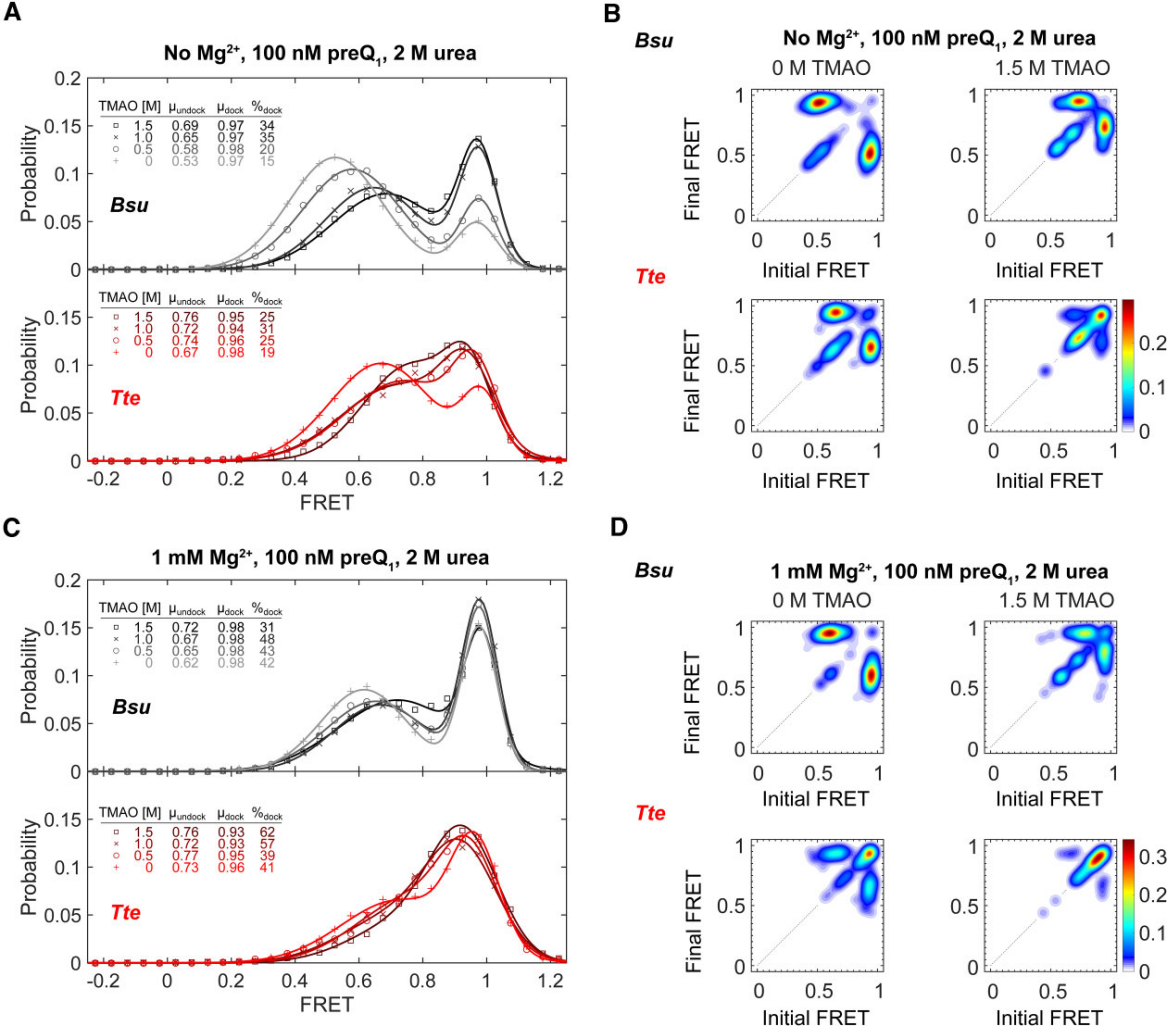
TMAO compacts the undocked state and stabilizes the folded conformation by counteracting urea-induced denaturation. (**A**) FRET population distribution histograms of the *Bsu* (top panel) and *Tte* (bottom panel) riboswitches in the absence of Mg^2+^ and presence of 2 M urea at increasing TMAO concentrations, showing shifts to a more compact mid-FRET state and a higher population of the high-FRET state. (**B**) Transition occupancy density plots (TODPs) for the *Bsu* (top) and *Tte* (bottom) riboswitches in the absence of Mg^2+^ and presence of 2 M urea, summarizing the FRET transitions in the absence and presence of 1.5 M TMAO. (**C**, **D**) FRET population distribution histograms and TODPs as in panels a and b, respectively, but with 1 mM Mg^2+^ added.100 nM preQ_1_ was present in all experiments to induce dynamic docking of the molecules.

We also investigated the ability of TMAO to counteract the denaturing effects of urea. In the presence of 2 M urea, TMAO titration results in a gradual shift in the μ_undock_ towards higher mid-FRET values for both the *Bsu* and *Tte* riboswitches, regardless of the presence or absence of 1 mM Mg^2+^ (Figure 5A, C). Notably, 1 M TMAO is able to counteract 2 M urea, almost restoring the fraction docked to the levels similar to those observed in the absence of urea (compare Figures 4 and 5). Furthermore, besides inducing overall compaction of both *Bsu* and *Tte* riboswitches, we observed an additional effect of TMAO on the *Tte* riboswitch. It shifts the dynamic transitional population towards a more highly folded static population, as highlighted by the TODPs at high TMAO concentration, which are dominated by the static high-FRET population (Figure 5B, D). Kinetic analysis shows that, in the absence of Mg^2+^, TMAO increases *k*_dock_ and decreases *k*_undock_, whereas in the presence of Mg^2+^, TMAO only increases *k*_dock_ without affecting *k*_undock_ (Supplementary Figure S4). Together, these observations suggest that TMAO promotes folding of both riboswitches, with the *Tte* riboswitch exhibiting a more compact overall conformation, as shown by the dominating static high-FRET population (Figure 5B, D). This highlights the higher tendency of the *Tte* riboswitch to fold.

### Osmolytes-induced changes in folding free energies

From the equilibrium constant of the folding, K_*eq*_, calculated from the area under the curve (AUC) for the population histograms showing mid- and high-FRET peaks using the equation K_*eq*_ = (*AUC*_*high FRET*_*/AUC*_*mid FRET*_) (used to be able to consistently capture both the dynamic and static docking behaviors we observe), osmolyte-dependent changes in Gibbs free energy of the folding process (ΔG°_*fold*_), known as *m-*values, were determined. ΔG°_*fold*_ was then derived using the Gibbs equation (ΔG°_*fold*_ = *–RTln*[K_*eq*_]). ΔG°_*fold*_ appears to be linearly correlated to osmolyte concentration, with the slope of a regression line defined as the *m-*value. This *m-*value analysis is commonly performed in osmolyte titration studies where changes in the *m-*values indicate the sensitivity of folding transitions to the presence of the osmolytes, reflecting the relative amount of solute biopolymer surface exposed to solvent upon unfolding (18).

**Figure 6.**
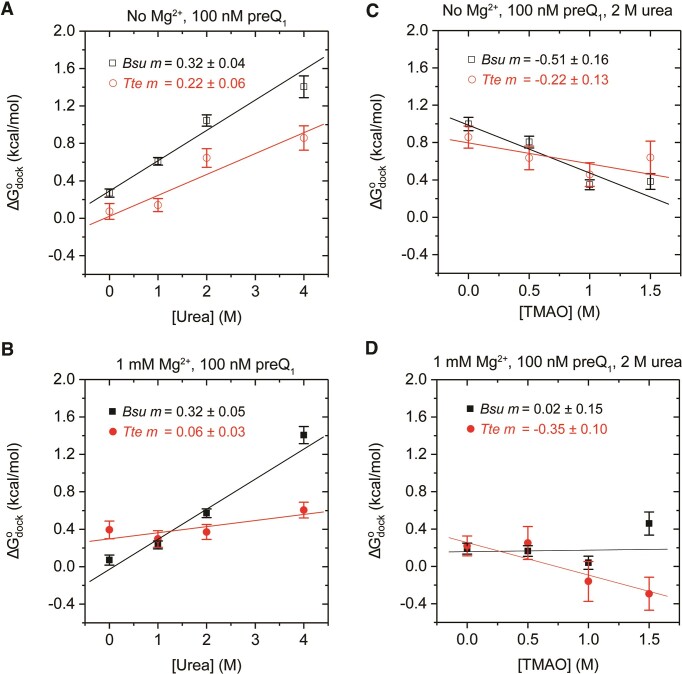
*m*-value analysis reveals distinct folding behaviors of the *Bsu* and *Tte* riboswitches. (A, B) Urea-dependent folding free-energy changes for *Bsu* (black) and *Tte* (red) riboswitches in the absence (**A**) and presence (**B**) of Mg^2+^. (**C**, **D**) TMAO-dependent folding free-energy changes for the *Bsu* (black) and *Tte* (red) riboswitches in the presence of urea and in the absence (C) and presence (D) of Mg^2+^. 100 nM preQ_1_ was present in all experiments. Data points in each panel originate from individual titrations from independent experiments performed on different slides. Error bars were derived from the standard deviations of fit coefficients from 1000 bootstrap replicates. The *R*-squared values for the linear fits of the *Bsu* and *Tte* data, respectively, are as follows: (A) 0.96, 0.86; (B) 0.95, 0.67; (C) 0.85, 0.58; (D) 0.01, 0.86.

In both the absence and presence of 1 mM Mg^2+^ under a 100 nM preQ_1_ background, the *Bsu* riboswitch shows the same degree of destabilization in response to increasing urea concentration, as indicated by the identical *m*-values of *m_-_*_Mg_^2+^ = 0.32 ± 0.04 kcal mol^−1^ M^−1^ and *m*_+Mg_^2+^ = 0.32 ± 0.05 kcal mol^−1^ M^−1^ (Figure 6a,b). In contrast, the *Tte* riboswitch is less susceptible to urea-induced denaturation, as evidenced by lower *m*-values (*m-*_Mg_^2+^ = 0.22 ± 0.06 kcal mol^−1^ M^−1^ and *m*_+Mg_^2+^ = 0.06 ± 0.03 kcal mol^−1^ M^−1^). These findings support our previous observation of a more tightly folded *Tte* riboswitch structure. Notably, the *m*-value of *Tte* in the presence of 1 mM Mg^2+^ approaches zero (*m*_+Mg_^2+^ = 0.06 ± 0.03 kcal mol^−1^ M^−1^), suggesting that *Tte* folding remains largely unaffected by urea. This observation is consistent with a Mg^2+^-stabilized, preQ_1_-bound structure for *Tte*, where little additional surface area becomes exposed to solvent upon the addition of urea.

In our TMAO titrations, the *m*-values for both riboswitches are close to zero, irrespective of the presence of Mg^2+^ (data not shown), suggesting minimal additional exposed surface area in the absence of TMAO compared to its presence. However, our previous observation of increasing mean mid-FRET values suggests an overall structural compaction. These two findings imply that structural compaction can occur without much solvent expulsion. Furthermore, we calculated *m*-values for TMAO’s effects on counteracting urea denaturation. Consistent with our previous results, we observed TMAO-induced stabilization of the both pseudoknots, as indicated by negative *m*-values for both riboswitches under all conditions, except for the *Bsu* riboswitch in the presence of both Mg^2+^ and preQ_1_, which showed an *m*-value close to zero (*m*_+Mg_^2+^ = 0.02 ± 0.15 kcal mol^−1^ M^−1^). This observation may be due to that the *Bsu* riboswitch is already stabilized by Mg^2+^ and then further reinforced by preQ_1_ binding. Consequently, this structure may exhibit resistance to additional TMAO-induced folding.

## Discussion

In this work, using smFRET microscopy coupled with UV-melting studies, we quantitatively compared the distinct kinetic and thermodynamic folding properties of the mesophilic *Bsu* and thermophilic *Tte* preQ_1_ riboswitch RNAs. We investigated their folding properties in the presence of cognate preQ_1_ ligand and the osmolytes urea and TMAO. Thermal denaturation experiments demonstrated that the higher-order structure of the *Bsu* riboswitch pseudoknot melts more cooperatively with a single broad transition, whereas *Tte* stems P2 and P1 melt in a stepwise fashion, unveiling a higher thermal stability of the *Tte* P1 helix in particular (Figure 2). This observation is consistent with the higher (80%) G-C content of P1 stem in *Tte* riboswitch compared to the *Bsu* riboswitch (60%), revealing one strategy for the evolutionary adaption of a functional RNA from meso- to thermophilic environmental conditions. Our smFRET-based chemical denaturation experiments further uncovered a higher structural stability for the *Tte* compared to the *Bsu* riboswitch. Urea denatures both *Bsu* and *Tte* riboswitches similarly by decreasing μ_undock_ and μ_dock_; however, *Tte* exhibits a greater resistance to urea-induced denaturation than the *Bsu* riboswitch (Figure 4), uncovering beneficial sequence changes in the *Tte* riboswitch for adaption to a high cellular urea content. Furthermore, we showed that TMAO has only a subtle effect on the conformation of the *Bsu* riboswitch in the absence of Mg^2+^, which is entirely lost upon addition of Mg^2+^. However, our data also demonstrated that TMAO plays a significant role in counteracting urea-induced structural destabilization by promoting the folded riboswitch conformation through increasing *k*_dock_ (Supplementary Figure S4). This indicates how high cellular urea concentrations can be counterbalanced by TMAO production.

Previous studies on urea denaturation have suggested that the addition of urea decreases the global stability of RNA, disrupting both secondary and tertiary structures (38). Numerous experimental and theoretical studies have investigated the mechanism underlying urea-induced denaturation of nucleic acids (39,40). These studies suggest that urea forms favorable interactions with nucleobases, sugars and phosphodiester backbones, which compete with internal RNA interactions (18). In bacteria, riboswitches have naturally evolved to express proteins that degrade or transport urea out of the cell in order to minimize its toxic effects (41). Driven by the underlying energetic forces, nucleic acids in the presence of urea tend to unfold to expose a larger solvent accessible surface area (SASA) and attract more urea molecules (42). Consistent with these studies, our results demonstrate that urea destabilizes both the *Bsu* and *Tte* riboswitches, shifting the population towards an unfolded conformation. Interestingly, the *m*-values for *Bsu* and *Tte* riboswitches are 0.32 and 0.22 (Figure 6), respectively, indicating that *Bsu* is more sensitive to urea and exposes relatively more SASA upon unfolding compared to *Tte*. This difference can be attributed to the longer dynamic loop L2 in the *Bsu* riboswitch, which is largely solvent exposed (Figure 1A). Furthermore, smFRET allowed us to probe the kinetic origin of urea-induced unfolding, which revealed a decrease in *k*_dock_ and an increase in *k*_undock_, with differential effects on the structurally similar *Bsu* and *Tte* riboswitches that are consistent with their mesophilic and thermophilic origins, respectively.

The zwitterionic TMAO also interacts with the nucleobases, sugars, and phosphodiester backbones of nucleic acids, but in an energetically unfavorable fashion. Therefore, exclusion of TMAO from the RNA surface stabilizes a more compact RNA conformation (28). Our data show that TMAO compensates for urea-induced denaturation of both riboswitches. Somewhat surprisingly, TMAO only seems to significantly counteract the effect of urea for the *Bsu* aptamer in the absence of Mg^2+^ (*m*-value –0.51 versus 0.02 in the absence and presence of Mg^2+^, respectively), whereas for the *Tte* riboswitch, the effects of TMAO and Mg^2+^ appear to be additive (Figure 6). This observation is consistent with TMAO promoting folding primarily through its unfavorable interactions with phosphates, whereas Mg^2+^ independently stabilizes folding by bridging two singly negatively charged backbone phosphates. In the case of the *Tte* riboswitch, additional compaction resulting in less phosphate exposure (as induced by TMAO) is nevertheless possible, as evidenced by the negative m-values as a function of TMAO concentration in the presence of 2 M urea. This conclusion is further corroborated by the higher fraction of high-FRET molecules observed for the *Tte* riboswitch than the *Bsu* riboswitch in TMAO, even in the presence of urea.

Finally, our *m-*value analysis shows that the *Tte* riboswitch has a higher tendency to remain folded than the *Bsu* riboswitch during osmolytic denaturation, consistent with our thermal denaturation studies. Since sensitivity to osmolytes is closely related to the SASA of a nucleic acid structure, our results suggest that the relative change in SASA for the *Bsu* riboswitch is higher than that for the *Tte* riboswitch. This agrees with the fact that the *Bsu* riboswitch has a 2-nucleotide larger loop L2 (Figure 1A) that is dynamic in the crystal structure and not as tightly folded as that of the *Tte* riboswitch (10,43–45). Such a dynamic and more open conformation of the loop may allow easy penetration of urea into the core of the RNA structure, thereby helping urea denature the *Bsu* riboswitch. Further support for this notion arises from the observation of a further modified L2 loop sequence in the psychrotolerant *Carnobacterium antarcticum* preQ_1_ riboswitch (from an organism growing at low temperatures in the Antarctic), which was recently found to be sufficiently expansive to accommodate two stacked ligand molecules (45). Based on our results, we anticipate that the preQ1 riboswitch from *Carnobacterium antarcticum* will demonstrate decreased thermal stability and heightened susceptibility to chemical denaturation due to a larger solvent exposure area. It is also known that several marine organisms such as sharks, which feature high cellular urea concentrations, naturally use a 1:2 ratio of TMAO:urea to counteract the denaturation stress of urea. In addition, several investigations have shown that TMAO can rescue proteins from urea-induced denaturation at this and other osmolyte ratios (24,46).

Our comparative study of two closely related riboswitches from a mesophilic and a thermophilic bacterium builds upon this line of inquiry and provides deep insights into the intricate interplay between RNA conformational dynamics and sequence adaptations in thermophilic organisms during their evolution from mesophilic organisms to thrive in new ecological niches.

## Supplementary Material

gkad866_Supplemental_File

## Data Availability

The various Matlab scripts, input data, and resulting analysis files associated with this work are available in DeepBlue from the University of Michigan library (https://doi.org/10.7302/hg74-pm89).
