# Supplementary material for: Single-molecule FRET observes opposing effects of urea and TMAO on structurally similar meso- and thermophilic riboswitch RNAs
Source: Nucleic Acids Res. 2023 Oct 19;51(20):11345–57. doi: 10.1093/nar/gkad866 (PMC10639078; doi:10.1093/nar/gkad866)
Supplement: gkad866_Supplemental_File [file gkad866_supplemental_file.pdf]

Supplementary Material

Single-molecule FRET observes opposing effects of urea and TMAO on structurally similar meso- and thermophilic riboswitch RNAs

Qian Hou[1,2], Surajit Chatterjee[1], Paul E. Lund[1], Krishna C. Suddala[1,3] and Nils G. Walter[1,*]

[1]**Single Molecule Analysis Group**, Department of Chemistry, University of Michigan, Ann Arbor, MI 48109, USA.

[2]**Present address:** Tri-Institutional PhD Program in Chemical Biology, Weill Cornell Medicine, The Rockefeller University, Memorial Sloan Kettering Cancer Center, New York, NY 10021, USA.

[3]**Present address:** Laboratory of Molecular Biology, National Institute of Diabetes and Digestive and Kidney Diseases, NIH, Bethesda, MD, 20892, USA.

*To whom correspondence should be addressed: nwalter@umich.edu

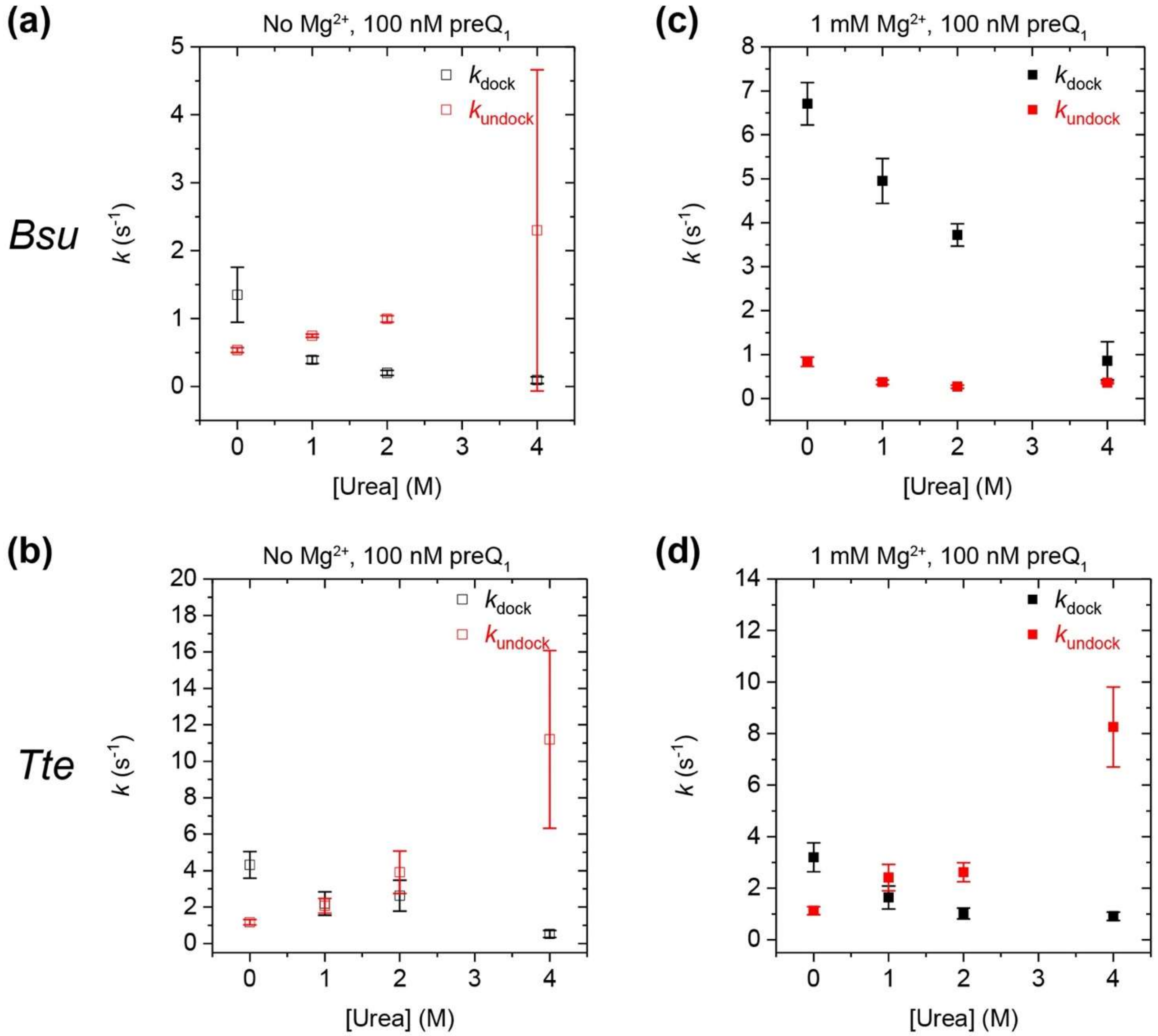

FIG. S1. Urea-dependent docking and undocking of the *Bsu* and *Tte* riboswitches. (a, c) Urea-dependent docking (k_{dock}) and undocking (k_{undock}) rate constants for the *Bsu* riboswitch in the absence (a) and presence (c) of Mg^{2+}. (b, d) Same as in panels a and c for *Tte* riboswitch. 100 nM $preQ_1$ was present in all experiments. The error was estimated by bootstrapping using a custom MATLAB script for fitting 1,000 bootstrap replicates. We note that the error bars in k_{undock} at 4 M urea are quite large in panels a and b. This results from the fact that very few molecules are observed to undergo changes in docking state under these conditions and so the rate is calculated from a relatively small number of events. Hence, the reported k_{undock} values should be considered as estimates only.

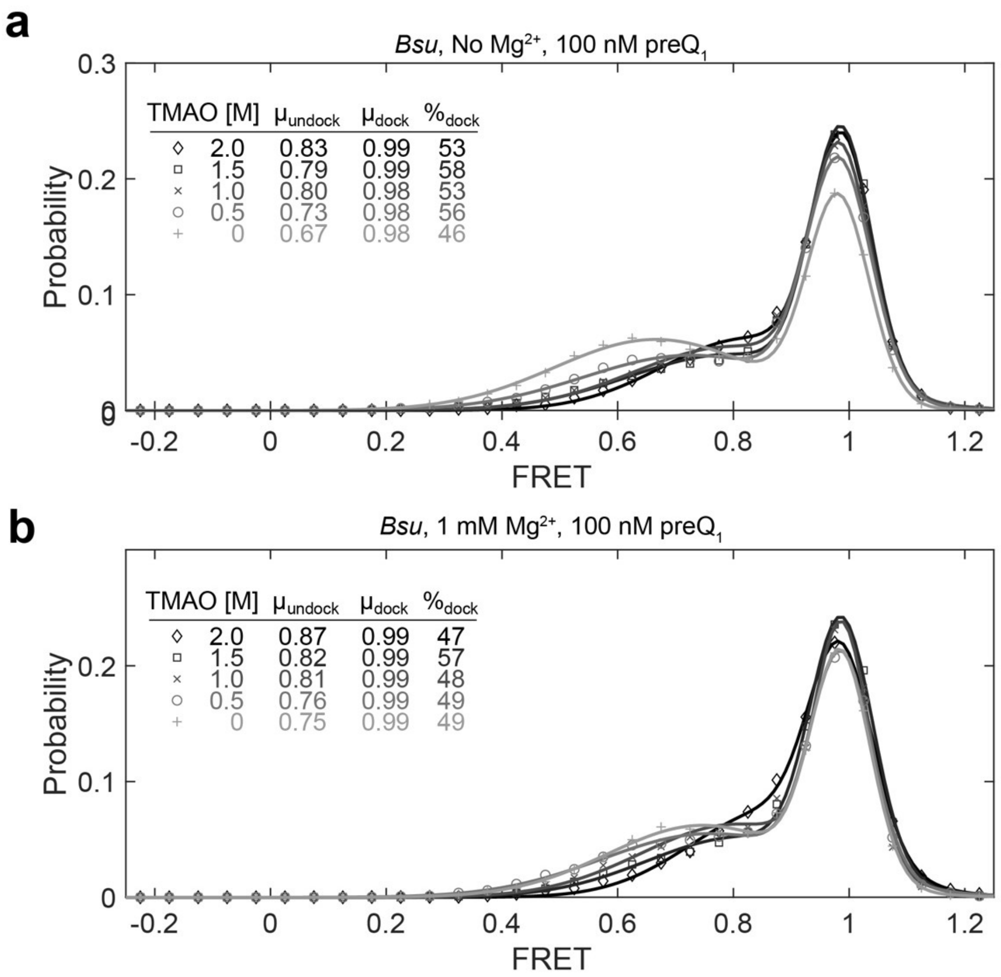

FIG. S2. Changes in the FRET population histograms of the *Bsu* riboswitch as a function of TMAO concentration in the absence (a) and presence (b) of Mg^{2+}. 100 nM $preQ_1$ was present in all experiments.

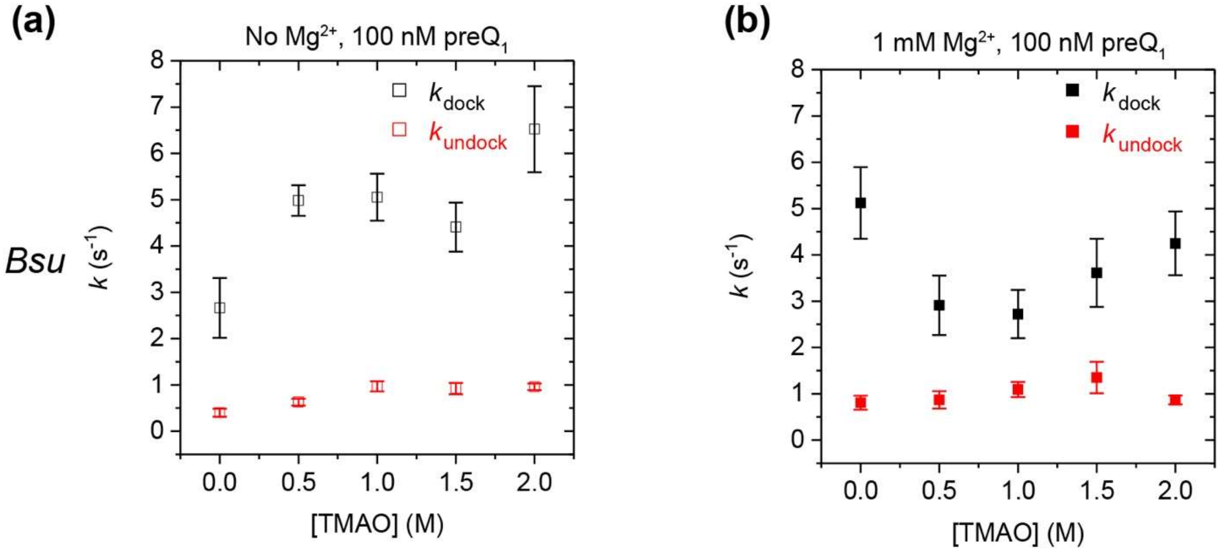

FIG. S3. TMAO-dependent docking and undocking of the Bsu riboswitch. TMAO-dependent docking (k_{dock}) and undocking (k_{undock}) rate constants for the *Bsu* riboswitch in the absence (a) and presence (b) of Mg^{2+}. 100 nM $preQ_1$ was present in all experiments. The error was estimated by bootstrapping using a custom MATLAB script for fitting 1,000 bootstrap replicates.

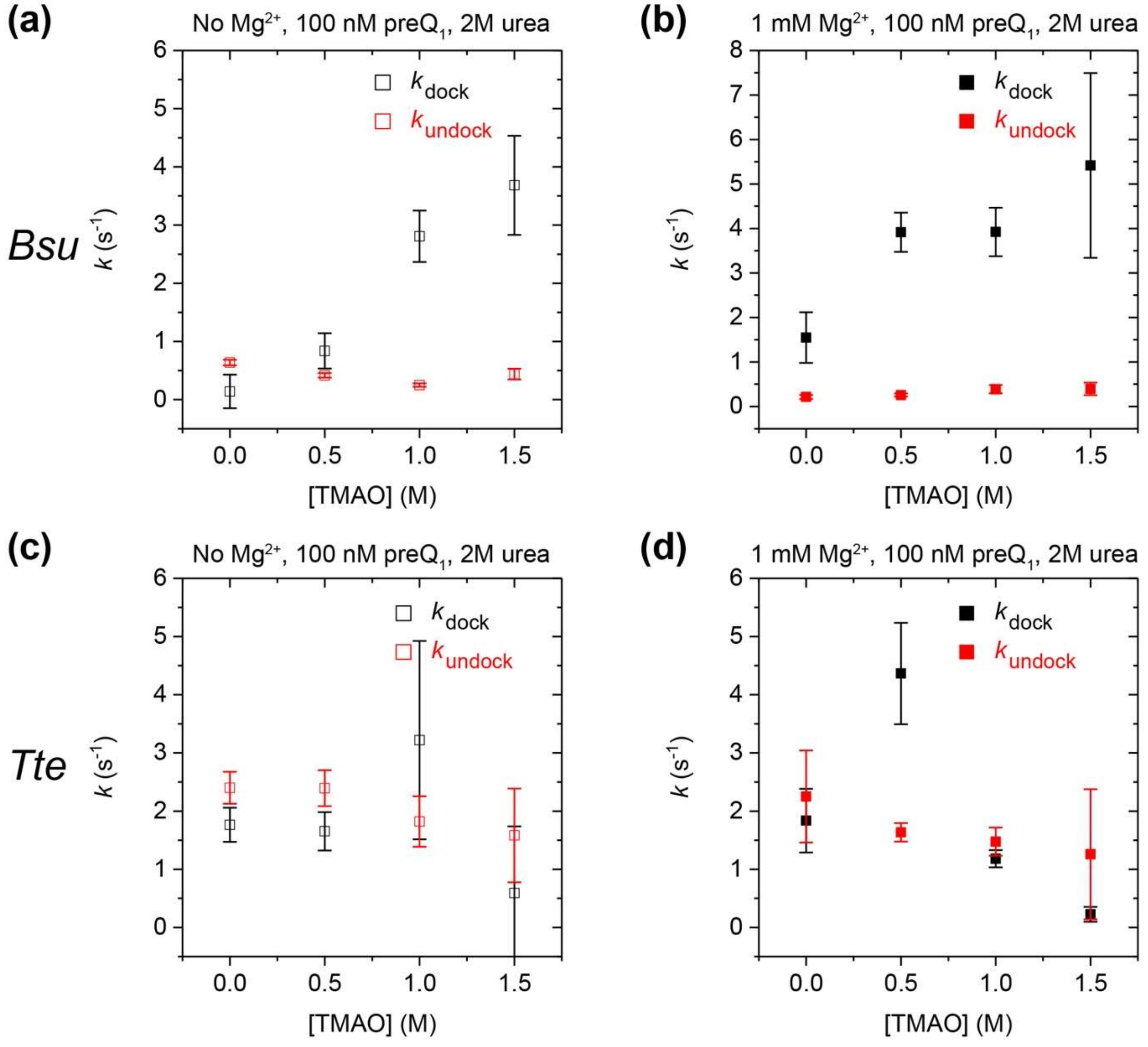

FIG. S4. TMAO-dependent docking and undocking of the *Bsu* and *Tte* riboswitches in a background of 2 M urea. (a, b) TMAO-dependent docking (k_{dock}) and undocking (k_{undock}) rate constants for the *Bsu* riboswitch in the presence of 2 M urea and in the absence (a) and presence (b) of Mg^{2+}. (c, d) Same as in panels a and b for the *Tte* riboswitch. 100 nM $preQ_1$ was present in all experiments. The error was estimated by bootstrapping using a custom MATLAB script for fitting 1,000 bootstrap replicates.

Condition	Riboswitch	Mg^{2+} (mM)	Urea (M)	TMAO	Number traces
01	*Bsu*	0	0	0	254
			1		363
			2		331
			4		262
02	*Bsu*	1	0	0	198
			1		293
			2		459
			4		363
03	*Tte*	0	0	0	97
			1		100
			2		106
			4		94
04	*Tte*	1	0	0	110
			1		112
			2		108
			4		120
05	*Bsu*	0	0	0.0	261
				0.5	266
				1.0	206
				1.5	174
				2.0	135
06	*Bsu*	1	0	0.0	143
				0.5	124
				1.0	116
				1.5	104
				2.0	93
07	*Bsu*	0	2	0.0	190
				0.5	176
				1.0	155
				1.5	94
08	*Bsu*	1	2	0.0	125
				0.5	152
				1.0	92
				1.5	67
09	*Tte*	0	2	0.0	81
				0.5	87
				1.0	82
				1.5	68
10	*Tte*	1	2	0.0	80
				0.5	71
				1.0	45
				1.5	64

Supplementary Table S1. Total number of molecules used for smFRET analysis under each experimental condition tested here.

Condition	Riboswitch	Mg^{2+} (mM)	Urea (M)	TMAO (M)	N kinetic traces	k(dock)	k(undock)
01	*Bsu*	0	0	0	94	double	double
			1		183	double	double
			2		135	double	single
			4		68	double	double
02	*Bsu*	1	0	0	50	double	double
			1		99	double	double
			2		133	double	double
			4		102	double	single
03	*Tte*	0	0	0	36	double	double
			1		35	double	double
			2		36	double	double
			4		21	double	double
04	*Tte*	1	0	0	29	double	double
			1		36	double	double
			2		32	double	double
			4		33	double	double
05	Bsu	0	0	0	100	double	double
				0.5	139	double	double
				1	74	double	double
				1.5	41	double	double
				2	27	double	single
06	Bsu	1	0	0	56	double	double
				0.5	38	double	double
				1	34	double	double
				1.5	68	double	double
				2	20	double	single
07	Bsu	0	2	0	136	double	double
				0.5	137	double	double
				1	104	double	single
				1.5	55	double	double
08	Bsu	1	2	0	98	double	double
				0.5	96	double	double
				1	55	double	double
				1.5	31	double	single
09	Tte	0	2	0	45	double	double
				0.5	25	double	single
				1	26	double	single
				1.5	15	single	single
10	Tte	1	2	0	23	double	double
				0.5	13	double	single
				1	10	double	single
				1.5	5	single	single

Supplementary Table S2. Summary of dynamic traces contributing dwell times to the rate constant determinations and whether single or double exponential fitting is used for each experimental condition.

PreQ1 riboswitch	[Urea] (M)	k_{dock} (s^{-1})	k_{undock} (s^{-1})	K_{dock}[a]	$\Delta\Delta G(K_{dock})$[b] (kcal mol^{-1})	$\Delta\Delta G^{\ddagger}(k_{dock})$[c] (kcal mol^{-1})	Φ[d]
Bsu	0	1.4	0.5	2.70	NA	NA	NA
	4	0.1	2.3	0.04	2.42	1.53	0.63
Tte	0	4.3	1.2	3.58	NA	NA	NA
	4	0.5	11.0	0.05	2.56	1.26	0.49

a. $K_{\text{dock}} = k_{\text{dock}}/k_{\text{undock}}$
b. $\Delta\Delta G(K_{\text{dock}}) = -RT\ln(K_{\text{dock}}^{\text{mut}}/K_{\text{dock}}^{\text{wt}})$
c. $\Delta\Delta G^{\ddagger}(k_{\text{dock}}) = -RT\ln(k_{\text{dock}}^{\text{mut}}/k_{\text{dock}}^{\text{wt}})$
d. $\Phi = \Delta\Delta G^{\ddagger}(k_{\text{dock}})/\Delta\Delta G(K_{\text{dock}})$

Supplementary Table S3. Calculations of transition-state analysis (Φ) values for *Bsu* and *Tte.*
